# Linezolid Inhibited Synthesis of ATP in Mitochondria: Based on GC-MS Metabolomics and HPLC Method

**DOI:** 10.1155/2018/3128270

**Published:** 2018-10-16

**Authors:** Xuemei Ye, Aifang Huang, Xianqin Wang, Congcong Wen, Lufeng Hu, Guanyang Lin

**Affiliations:** ^1^Department of Pharmacy, The First Affiliated Hospital of Wenzhou Medical University, Wenzhou 325000, China; ^2^Laboratory Animal Center, Wenzhou Medical University, Wenzhou 325035, China

## Abstract

Linezolid has been widely used in serious infections for its effective inhibiting effect against multidrug-resistant gram-positive pathogens. However, linezolid caused severe adverse reactions, such as thrombocytopenia, anaemia, optic neuropathy, and near-fatal serotonin syndrome. In order to investigate the toxicity of linezolid, twenty-four Sprague-Dawley rats were randomly divided into: control group (n=7), low-group (n=8), and high-group (n=9). The rats of low-group and high-group were given by gavage with linezolid 60 and 120 mg/kg/day for 7 days, respectively. The serum concentration of linezolid was determined by high performance liquid chromatography (HPLC); blood metabolic change was analyzed by gas chromatography-mass spectrometer (GC-MS). Adenosine triphosphate (ATP) concentration in HepG2-C3A after being cultured with linezolid was determined by HPLC. The results showed that there were six metabolites and nine metabolites had statistical differences in low-group and high-group (P<0.05). The trimethyl phosphate was the most significant indicator in those changed metabolites. Except for d-glucose which was slightly increased in low-group, octadecanoic acid, cholest-5-ene, hexadecanoic acid, *α*-linolenic acid, eicosapentaenoic acid, 9,12-Octadecadienoic acid, and docosahexaenoic acid were all decreased in low-group and high-group. ATP concentration was decreased in HepG2-C3A after cultured with linezolid. In conclusion, the toxicity of linezolid is related to its serum concentration. Linezolid may inhibit the synthesis of ATP and fatty acid.

## 1. Introduction

Linezolid, the first approved synthetic oxazolidinone, is active against multidrug-resistant gram-positive pathogens, including streptococci, vancomycin-resistant enterococci (VRE), and methicillin-resistant* staphylococcus aureus* (MRSA), several anaerobes,* Nocardia* species, and some mycobacteria [1–4]. Linezolid has a lower mortality than daptomycin in treatment for VRE bacteremia [5]. It is a viable option in the treatment of multidrug-resistant or extensively drug-resistant tuberculosis [6]. Moreover, bacterial resistance to linezolid has remained very low since it was approved by the US Food and Drug Administration (FDA) on the market in 2000 [7]. Therefore, linezolid has been used as a very effective antibacterial in some serious infections as a first-line medicine [8, 9].

However, linezolid caused severe adverse reactions. The haematological toxicity is the most common and main toxic reaction, characterized particularly by thrombocytopenia (with an incidence at 32%-45.8%) and anaemia (with an incidence at 25%) [10–12]. The haematological adverse reaction happened always in patients with cancer who had poor marrow reserve. In addition, linezolid induced toxic optic neuropathy [13], near-fatal serotonin syndrome [14], and acute interstitial nephritis [8]. Those toxic effects always occur more frequently in patients who receive linezolid for two weeks or more long time. The mechanism study showed that those toxic reactions were probably immune-mediated [15]. However, the exact mechanism of its toxicity still remains unclear.

Metabonomics is a useful tool in toxic analysis as it provides a unique mechanistic perspective on responses to toxicity [16–19]. In recent years, it has been widely applied to investigate the systematic metabolic responses to toxins [20] and associated mechanisms [21]. To date, there is no study focus on the toxicity response of linezolid by the use of metabonomics technology. In this study, we firstly investigated the effect of linezolid on blood metabolic changes in rat by gas chromatography-mass spectrometer (GC-MS).

## 2. Methods

### 2.1. Drug and Reagents

Linezolid (purity > 98%) was provided by Fresenius Kabi Norge AS (Norway). Adenosine triphosphate (ATP) (purity > 95%) was purchased from Dalian Meilun Biotechnology Co., Ltd. The derivatization reagents, N-Methyl-N-(trimethylsilyl) trifluoroacetamide and trimethylchlorosilane (TMCS), were both purchased from Sigma-Aldrich in Shanghai China, while methylhydroxylamine hydrochloride and pyridine were from Aladdin Industrial, Inc. (Shanghai, China). The other HPLC-grade acetonitrile and n-heptane were purchased from Tedia Reagent Company (Shanghai, China).

### 2.2. Instruments

An Agilent GC/MS (6890N-5975B, Santa Clara, California, USA) and HP-5MS (0.25 mm×30 m×0.25 mm) were used for metabonomics study. The determination condition was set as follows: firstly, the GC oven was set at 80°C and kept for 5 min. Then, the temperature was increased to 260°C at a rate 10°C/min and kept at 260°C for 10 minutes. MS detection was conducted in EI mode with electron energy of 70 eV, and then in full-scan mode with m/z of 50-550.

An Agilent 1260 Infinity HPLC system 1260 which included an online degasser, quaternary pump, autosampler, thermostat-columned compartment, and diode-array UV detector was used for determination of linezolid concentration.

### 2.3. Animal and Cell

Twenty-four Sprague-Dawley rats (male, 220±20 g) were purchased from Shanghai SLAC Laboratory Animal Co., Ltd. Animals were used in this study at laboratory animal research center of Wenzhou Medical University. All of experimental procedures were approved by Experimental Animals Administration Committee of Wenzhou Medical University.

HepG2-C3A cell was a gift from First Affiliated Hospital, College of Medicine, Zhejiang University. The HepG2-C3A cell was slowly added into a culture bottle by 1:10 dilution ratio after being thawed at room temperature. After that, HepG2-C3A was cultured in CO2 incubator, at saturated humidity, 37°C and 5% CO2.

### 2.4. Development of HPLC Method of Linezolid

The linezolid were separated by HC-C18 column (Agilent, 2.1 mm × 150 mm, 5 *μ*m) with temperature set at 30°C, detection wavelength at 258 nm, and isocratic elution composed of mobile phase A (acetonitrile, 13%) and mobile phase B (87%) at a flow rate of 1 mL/min. The mobile phase B prepared with 5 mM potassium dihydrogen phosphate and adjusted to PH 2.3 by phosphoric acid.

The 100 *μ*L perchloric acid-methyl alcohol (1:9) was added into 200 *μ*L serum and vortex mixed for 0.5 min, after that it centrifuged at 10,000 g for 10 minutes. The supernatant (20 *μ*L) was injected into the HPLC system for analysis.

The concentration of linezolid was calculated with the calibration curves which were constructed at eight calibration standards samples (0.125, 0.25, 0.5, 1, 2, 4, 8, and 16 *μ*g/mL). The developed determination method has been comprehensively validated in the intraday precision, interday precision, and accuracy.

### 2.5. Development of HPLC Method of ATP

The ATP was separated by Polaris 5 C18-A column (Agilent, 2.1 mm × 150 mm, 5 *μ*m) with temperature set at 25°C, detection wavelength at 258 nm, and isocratic elution composed of mobile phase A (methanol, 0.6%) and mobile phase B (99.4%) at a flow rate of 1 mL/min. The mobile phase B prepared with 25 mM sodium dihydrogen phosphate and adjusted to PH 7.0 by triethylamine.

The 40 *μ*L 25% perchloric acid was added into 200 *μ*L culture solution and vortex mixed for 0.5 min, after that it was centrifuged at 10,000 g for 10 minutes. The supernatant (20 *μ*L) was injected into the HPLC system for analysis.

The concentration of ATP was calculated with the calibration curves which were constructed at six calibration standards samples (24, 12, 6, 3, 1.5, and 0.75 *μ*g/mL). The developed determination method has been comprehensively validated in the intraday precision, interday precision, and accuracy. The stability study was carried out at 0, 6, 12, and 24 hours at room temperature.

### 2.6. Animal Study

Twenty-four rats were randomly divided into three groups, control group (n=7), low-group (n=8), and high-group (n=9). Before the experiment, the rats were housed under a natural light-dark cycle conditions with controlled temperature (22°C).

The rats of low-group and high-group were given by gavage with linezolid 60 and 120 mg/kg twice a day, respectively; the rats of control group were given by saline. At 9:00 am on day 8, after intragastric administration linezolid for 7 days, the blood samples of rats in three groups were collected from the caudal vein and centrifuged at 8500 rpm for 10 min. After that, the serum was separated and stored at -80°C.

The metabonomics was analyzed by GC-MS. The serum concentrations of linezolid and ATP were determined by high performance liquid chromatography (HPLC). The hematotoxicity of linezolid was evaluated by blood routine examination, which was determined by an automated hematology auto analyzer (Beckman, USA).

### 2.7. GC-MS Metabonomics

A total 100 *μ*L serum samples were added into a 1.5 mL tapered plastic centrifuge tubes kept at an ice-bath, and then 250 *μ*L acetonitrile was added. After being kept for 15 minutes, it was centrifuged at 10,000 g for 10 minutes at 4°C. After centrifugation; 150 *μ*L supernatant was drawn into a GC vial and evaporated under nitrogen gas. Later then, 50 *μ*L of methylhydroxylamine hydrochloride (15 mg/mL in pyridine) was added and carried out at 70°C for 24 hours. Subsequently, the catalyst (50 *μ*L MSTFA with 1% TMCS) was added for catalyzing reaction. After being catalyzed at 70°C for another hour, n-heptane 150 *μ*L was added and whirled for 1 minute; then the supernatant was separated and determined at GC-MS.

The quality control (QC) strategy was applied to monitor the variability within analytical batch and ensure the data quality as published articles [22, 23]. QC sample was prepared by equally mixing the serum from linezolid and control group, and QCs were processed together with samples by using the same method.

### 2.8. Cell Study

After continuous generation of HepG2-C3A cell, they were added into the 96-well plate according to the cell count, 1.8 ×10^4^ cells/well. When the HepG2-C3A cells adhered to the cell wall, 1 mg/mL 50uL linezolid were added and cultured in CO2 incubator, at saturated humidity, 37°C and 5% CO2. After 24 hours, HepG2-C3A cells were digested with pancreatin and centrifuged at 5,000 g for 10 minutes at 4°C. A total of 200 *μ*L cell culture medium containing HepG2-C3A cells were separated and precipitated by 40 *μ*L perchloric acid. After vortex was mixed for 0.5 min, it was centrifuged at 10,000 g for 10 minutes. The supernatant (20 *μ*L) was injected into the HPLC system for analysis.

### 2.9. Data Processing

Before multivariate analysis, the GC/MS peaks of metabolites were normalized to the total sum of spectrum. The peaks of metabolites detected by GC/MS were normalized and recorded in Microsoft Excel. After that, the principal component analysis (PCA) and partial least squares discriminate analysis (PLS-DA) were used to identify the differences of serum composition in two different groups by using SIMCA-P 11.5 software (Umetrics, Umea, Sweden). The variables contributing to the separation of samples on the scores plot were identified by corresponding loading plots.

Significantly changed metabolites were picked out according to the variable importance for project values (VIP > 1) and identified by similarity searches in the NIST 2005 mass spectral library. Independent samples t-test, conducted by using SPSS software (Version 13.0, SPSS), was applied to analyze the significant differences of metabolites between two groups.

## 3. Results

### 3.1. HPLC Method of Linezolid

**Figure 1:**
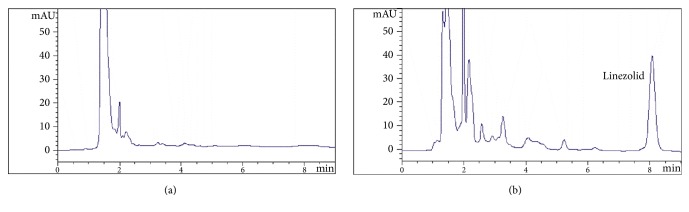
Typical HPLC chromatograms of linezolid (1, retention time is 8.05 min): (a) blank serum; (b) blank serum+8 *μ*g/mL linezolid.

Linezolid was separated well in the HC-C_18_ column and no interference was observed in the HPLC chromatograms. The retention time of linezolid was 8.05 min. The typical HPLC chromatograms were shown in Figure 1.

The calibration curves of linezolid were y = 43.72x - 0.205, which had good linearity (R^2^ = 0.999) in the range of 0.125-16.0 *μ*g/mL. The limit of detection was 0.05 *μ*g/mL when S/N was 1/10. The validation of this method showed that the relative standard deviations (RSD) of each QC sample (0.5, 5, and 15 *μ*g/mL) for intraday and interday precision were all less than 10%; the recovery of linezolid was higher than 70% (Table 1).

### 3.2. HPLC Method of ATP

**Figure 2:**
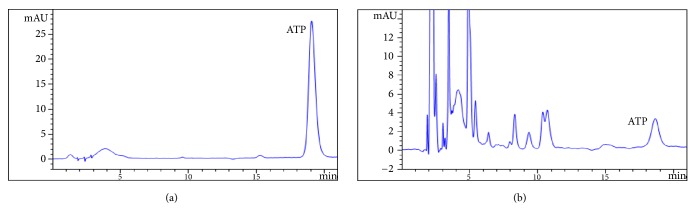
Typical HPLC chromatograms of ATP (retention time is 16.89 min). (a) Stand ATP 20 ug/mL; (b) HepG2-C3A cell sample.

ATP was separated well in the Polaris HepG2-C18-A column and no interference was observed in the HPLC chromatograms. The retention time of ATP was 16.8 min. The typical HPLC chromatograms were shown in Figure 2.

The calibration curves of ATP were y = 27.378x - 2.3467, R^2^ = 0.9996, which had good linearity in the range of 0.75-16.0 *μ*g/mL. The limit of detection was 0.3 *μ*g/mL when S/N was 1/10. The validation of this method showed that the relative standard deviations (RSD) of each QC sample (1.4, 4.2, and 12.6 *μ*g/mL) for intraday and interday precision were all less than 10%. The recovery of ATP at 1.4, 4.2, and 12.6 *μ*g/mL was 81.56±3.31%, 84.51±2.62%, and 83.26±1.51%, and mean recovery was 83.11±31.84%. The stability experiment of ATP showed it gradually degraded in room temperature (Table 2). Therefore, the cell samples showed to be determined quickly within 1 hour.

### 3.3. Linezolid Concentration and Metabolic Changes in Rat

#### 3.3.1. Serum Concentration of Linezolid

Based on the developed method, the serum concentration of linezolid in low-group and high-group was determined. The results showed that the serum concentrations of linezolid were all higher than 0.125 *μ*g/mL and the mean serum concentration was 0.39 ± 0.18 *μ*g/mL, in high-group. However, in low-group, only two rats were higher than 0.125 *μ*g/mL. The detailed serum concentration of linezolid in low-group and high-groups after intragastric administration for 7 days was shown in supplemented Table 1.

#### 3.3.2. Metabolic Changes in Blood

**Figure 3:**
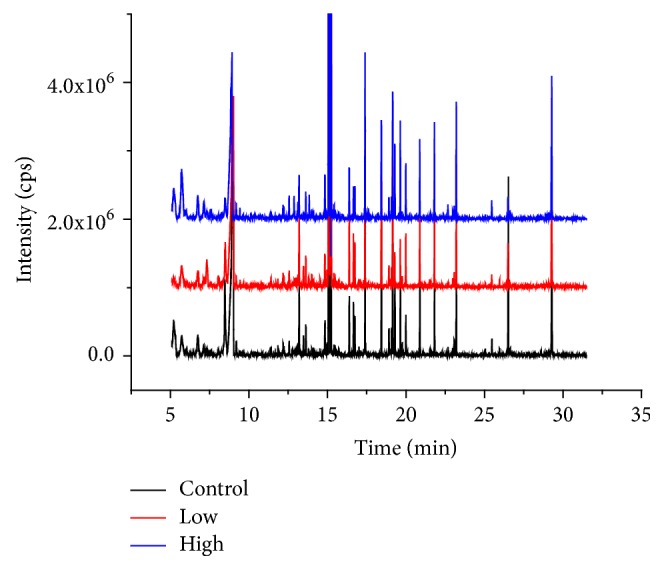
Typical GC-MS total ion chromatogram of rat serum in three groups (control, low, and high) after intragastric administration of linezolid.

The quality control samples were investigated to validate the reproducibility of the metabolic features before the sample analysis. The typical serum metabolic profiles of linezolid acquired through GC-MS technique were shown in Figure 3. There were 99 metabolic features identified from GC-MS analyses after normalization processing of metabolic data.

**Figure 4:**
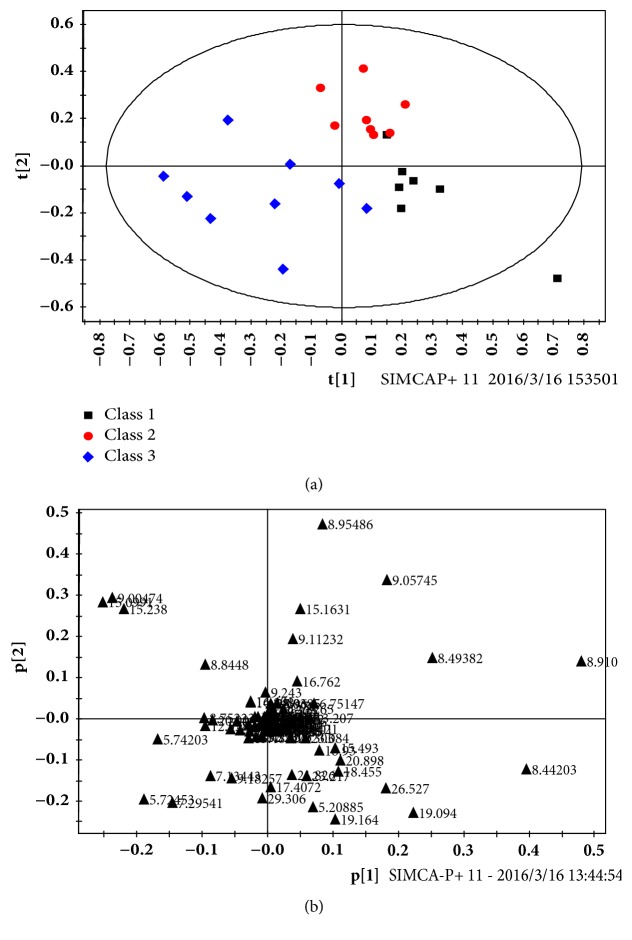
PLS-DA score results of rat serum samples: (a) after intragastric administration of linezolid (60, 120 mg/kg, Low, High), control (Class 1), low (Class 2), and high (Class 3); the corresponding load diagram (b). R2Y(cum) = 0.611 and Q2Y(cum) = 0.223.

According to the data extracted from GC-MS spectrum, PCA analysis showed that the high-group was completely separated with control-group. However, in low-group, there were several samples overlapped with control-group; the PCA score plots and load diagram figure were shown in supplement Figure 1. In order to obtain more accurate information, PLS-DA was used to analyze the GC-MS metabolic data. The score plots and corresponding load diagram ofPLS-DA were shown in Figure 4. The PLS-DA 2D (Figure 4(b)) score chart showed that the rats in low-group, high-group, and Control-group were all completely distinguished from each other.

According to the results of PLS-DA, there were 16 metabolites (VIP>1) selected in three groups, and independent samples t-test analysis was used for statistical analysis. The results showed that there were 11 metabolites that had statistical differences; the summary of the changed metabolites was listed in Table 3.

### 3.4. ATP Concentration in Rat

After 1 mg/mL, 50 *μ*L linezolid was added and cultured in CO2 incubator for 24 hours; HepG2-C3A cells were collected from 12-well plate and determined by developed HPLC method. The ATP concentration was 2.26±0.50 cultured with linezolid g/mL after being cultured with linezolid which was lower than 5.13±0.16 *μ*g/mL (P<0.05) in control cell without being linezolid. The detailed ATP concentrations of HepG2-C3A cells cultured with linezolid were shown in supplemented Table 2.

## 4. Discussions

In this study, the hematotoxicity of linezolid was evaluated by blood routine examination. The results (supplement Table 3) showed that the levels of white blood corpuscles, hemoglobin, hematocrit, mean corpuscular volume, mean corpuscular hemoglobin, mean corpuscular hemoglobin concentration, and platelets were all decreased in low and high group, which had statistic differences (P<0.05) compared with the control group.

Based on the serum metabolite and linezolid concentration in serum, the toxicity of linezolid was further studied. In high-group, there were nine metabolites had statistical differences, while there were six metabolites in low-group. As more blood metabolites had statistical significance in high-group, the toxicity of linezolid is related to its serum concentration. Among these metabolites, the level of trimethyl phosphate was decreased. Trimethyl phosphate is the trimethyl ester of phosphoric acid. Phosphoric acid and its derivative phosphates are nutritious for our life. They are found pervasively in our body, such as deoxyribonucleic acid (DNA), ribonucleic acid (RNA), and adenosine triphosphate (ATP); both of these compounds are derived from phosphorylated sugars [24, 25]. The total amount of DNA and RNA usually are remaining constant. Therefore, the decreased concentration of phosphoric acid may relate to the amount of ATP decreased.

Octadecanoic acid, hexadecanoic acid, *α*-Linolenic acid, eicosapentaenoic acid, and 9,12-Octadecadienoic acid all belong to fatty acids [26]. These fatty acids are usually derived from triglycerides or phospholipids and need adenosine triphosphate (ATP). The decreased level of these fatty acids indicated that there were lack triglycerides or phospholipids or ATP. Considering the decreased amount of trimethyl phosphate, the most possible reason was the amount of ATP decreased.

In order to confirm the results of GC-MS, an ATP determination method was developed by HPLC. Since the serum concentration of ATP was lower than the lowest detection limit (LOD), we evaluated the ATP concentration in HepG2-C3A cell. The result showed that ATP concentration decreased when cultured with linezolid.

ATP is a triphosphate, a small molecule used by enzymes in many cellular processes as intracellular energy transfer unit. It transports chemical energy for various biological activities, such as metabolism of exogenous and endogenous material, synthesis of fats, and proteins and sugars. Every day, the energy used by human cells requires 100 to 150 moles of ATP [27].

Substrate-level phosphorylation and oxidative phosphorylation are two major mechanisms of ATP biosynthesis. In aerobic conditions, one molecule of glucose is degraded into two molecules of pyruvic acid (pyruvate); two net molecules of ATP will be generated during the process. The reaction can be expressed this way:

  Glucose+2NAD^+^+2ADP+2Pi → 2 pyruvic acid +2ATP+2NADH+2H^+^+2H_2_O

Pyruvic acid goes into the mitochondria and is oxidized to acetyl-CoA and CO2 and then acetyl-CoA takes oxidative phosphorylation by Krebs cycle (tricarboxylic acid cycle). During the cycle, 3 NADH, 1 FADH2, and 1 GTP will be generated. NADH and FADH will establish a proton gradient and drive the phosphorylation of ADP to synthesize ATP by the ATP synthase enzyme. This process relied on the normal function of electron transport chain.

The electron transport chain is a series of compounds that transfer electrons from electron donors to electron acceptors, composed of complex I (NADH coenzyme Q reductase), complex II (succinate dehydrogenase), complex III (cytochrome bc1 complex), and complex IV (cytochrome c oxidase). Linezolid can inhibit bacterial protein synthesis by preventing the fusion of 30S and 50S ribosomal subunits [28]. Since some complex IV proteins are synthesized by mitochondrial ribosomes, linezolid probably interferes with mitochondrial protein synthesis which is similar to bacterial protein synthesis [29].

**Figure 5:**
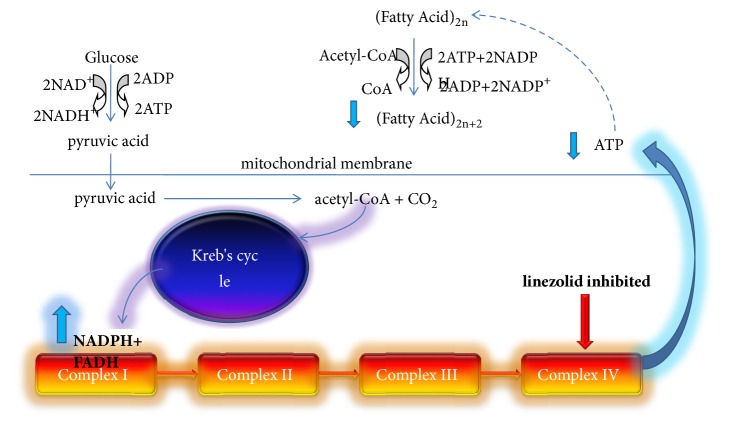
The possible mechanism of linezolid on the metabolism change in rat, increased glucose, and decreased ATP and fatty acid.

Therefore, the toxicity mechanism of linezolid may well be the function of electron transport chain damaged by the inhibition of complex IV proteins, which caused NADH and FADH cannot establish a proton gradient and synthesize ATP. More and more NADH and FADH cannot be transferred in electron transport chain; they would be accumulated in mitochondria, which would cause feedback inhibition of Krebs cycle, generation of pyruvic acid, and decomposition of glucose. This feedback inhibition would further reduce the amount of ATP. The possible toxicity mechanism of linezolid was shown in Figure 5.

It has been reported that linezolid caused hypoglycemia, lactic acidosis, and acute pancreatitis [28, 30]. Based on our study, the hypoglycemia and lactic acidosis may well be the subsequent damage of ATP energy metabolism. In the early stage, there will be a slight inhibition of glucose metabolism, when body still can produce a few of ATP to maintain life activities. That why the increased level of d-glucose only appeared in low-group but not high-group.

When the electron transport chain was totally inhibited, the body must produce ATP by substrate-level phosphorylation but not oxidative phosphorylation. The substrate-level phosphorylation will consume a large number of glucose and produce a plenty of pyruvic acid. The pyruvic acid will be transferred to lactic acid by lactic dehydrogenase, which will lead to lactic acidosis.

## 5. Conclusion

According to GC-MS based metabonomics study, trimethyl phosphate was the most significant indicator among changed metabolite after administration of linezolid. Except for d-glucose which was slightly increased in low-group, octadecanoic acid, cholest-5-ene, hexadecanoic acid, *α*-Linolenic acid, eicosapentaenoic acid, 9,12-Octadecadienoic acid, and docosahexaenoic acid were all decreased. The toxicity of linezolid is related to its serum concentration. The linezolid inhibits the synthesis of ATP in mitochondria, which decreased the synthesis of fatty acid. The energy metabolism should be monitored when the patients received high dose of linezolid.

## Figures and Tables

**Table 1:** Precision and recovery of method for the determination of linezolid in rat serum (n = 5).

Concentration (*μ*g/mL)	Intra-day		Inter-day		Recovery (%)
	Mean±SD	RSD	Mean±SD	RSD	
0.5	0.49±0.03	5.59	0.50±0.04	8.01	77.58±3.93
5	5.08±0.29	5.80	5.07±0.31	6.03	75.44±3.34
15	14.51±1.05	7.23	14.92±0.77	5.18	75.17±3.21

**Table 2:** The stability experiment of ATP in room temperature.

Time(h)	1.4 ug/mL		4.2 ug/mL		12.6 *μ*g/mL	
	Area	(%)	Area	(%)	Area	(%)
0.0	36.6	100.0	108.3	100.0	352.1	100.0
1.0	35.9	98.1	106.0	97.9	344.2	97.8
6.0	25.7	70.2	81.5	75.3	286.5	81.4
12.0	16.8	45.9	60.7	56.0	230.2	65.4
24.0	11.6	31.7	44.1	40.7	190.2	54.0

**Table 3:** The blood metabolic changes in control-group (1), low-group (2), and high- group (3) after administration of linezolid with two different dosages.

No	VIP		Control-group	Low-group	High-group	1v2	1v3
		Compound	Mean±SD	Mean±SD	Mean±SD	P	P
1	3.01	Trimethyl phosphate	5±2.3	3.3±0.2	1.7±0.9	0.189	0.007
2	2.87	Urea	3.5±0.3	4.9±2.7	2.4±1.8	0.338	0.243
3	2.62	carbonate	3.2±1.8	5.5±4.3	5.1±1.2	0.368	0.244
4	1.95	Octadecanoic acid	2.7±0.9	1.8±0.2	2.3±0.4	0.013	0.256
5	1.82	d-Mannose	0.2±0	1.3±1. 9	0.1±0	0.496	0.035
6	1.73	d-Glucose	9.4±0.8	10.7±0.8	10.7±2.7	0.01	0.248
7	1.67	Cholest-5-ene	2.1±1	1.1±0.7	0.9±1.1	0.046	0.046
8	1.65	Hexadecanoic acid	2.5±0.4	2.3±0.5	1.8±0.2	0.378	0.001
9	1.51	l-Methionine	3.4±0.4	2.9±0.2	3.5±0.7	0.005	0.783
10	1.24	Butanoic acid	1.9±1.7	1.1±1.1	0.9±0.7	0.49	0.197
11	1.08	*α*-Linolenic acid	1.7±0.5	1.5±0.8	1.1±0.2	0.586	0.011
12	1.08	Arabinofuranose	0.6±0.1	0.6±0.1	0.4±0.1	0.076	0
13	1.06	Eicosapentaenoic acid	1.6±0.4	1.2±0.2	1.2±0.3	0.039	0.028
14	1.05	9,12-Octadecadienoic acid	1±0.2	0.6±0.2	0.6±0.3	0.002	0.009
15	1.05	Docosahexaenoic acid	1.5±0.2	1.4±0.3	1.2±0.1	0.487	0.002
16	1.00	Retinoic acid, methyl ester	1.9±0.7	1.5±0.2	1.5±0.4	0.126	0.13

## Data Availability

Some data has been provided in supplemental table and figure.

## Supplementary Materials

Supplementary Materials Supplement Tables 1 and 2 listed the concentration of linezolid in rat and ATP in C3A cells. Supplement Table 3 listed indexes of blood routine test after administration of linezolid. Supplement Figure 1 showed the results of PCA analysis based on the GC-MS.
