# Supplementary material for: Linezolid Inhibited Synthesis of ATP in Mitochondria: Based on GC-MS Metabolomics and HPLC Method
Source: Biomed Res Int. 2018 Oct 16;2018:3128270. doi: 10.1155/2018/3128270 (PMC6206563; doi:10.1155/2018/3128270)
Supplement: Supplementary Materials — Supplement Tables 1 and 2 listed the concentration of linezolid in rat and ATP in C3A cells. Supplement Table 3 listed indexes of blood routine test after administration of linezolid. Supplement Figure 1 showed the results of PCA analysis based on the GC-MS. [file 3128270.f1.zip › 3128270.f1.pdf]

Supplement tables

Table 1. The serum concentration of linezolid (μg/mL) in Low-group and High-groups after intragastric administration for 7 days

NO.	High-group	Low-group
1.00	0.514	<0.125
2.00	0.830	<0.125
3.00	0.337	<0.125
4.00	0.298	<0.125
5.00	0.265	<0.125
6.00	0.389	<0.125
7.00	0.277	<0.125
8.00	0.406	0.232
9.00	0.211	0.154

Table 2. The detailed ATP concentration(μg/mL) of C3A cells cultured with linezolid

NO.	With linezolid	Without linezolid
1.00	2.843	4.974
2.00	1.976	5.302
3.00	1.970	5.138

Table 3. The blood parameters of three groups and ANOVA statistical analysis

Parameter	abbr	unit	Control-group	Low-group	High-group	Sig.
White blood corpuscles	WBC	109/L	6.73±1.39	6.26±1.22	5.19±1.29	0.038
Percentage of neutrophils	PN	%	40.93±13.78	41.06±4.19	39.64±9.86	0.941
Absolute neutrophil count	NC	109/L	3.07±1.42	2.20±0.58	2.37±0.59	0.115
Red blood corpuscles	RBC	1012/L	8.90±1.54	8.21±0.80	7.56±1.88	0.148
Hemoglobin	HGB	g/L	163.37±9.79	150.39±8.31	141.45±15.05	0.001
Hematocrit	HCT	%	49.35±6.88	46.15±5.76	40.16±8.38	0.023
Mean corpuscular volume	MCV	fl	71.54±16.54	59.87±6.40	57.15±6.57	0.015
Mean corpuscular hemoglobin	MCH	pg	21.82±5.04	18.28±1.93	17.43±2.00	0.015
Mean corpuscular hemoglobin concentration	MCHC	g/L	349.32±76.79	300.10±32.57	284.80±32.73	0.025
Platelets	PLT	109/L	584.4±129.64	447.6±118.51	436.8±113.02	0.018
